# Internal and external self-affirmation resources: validation and assessment of psychometric properties of the spontaneous self-affirmation measure using structural equation modeling

**DOI:** 10.3389/fpsyg.2024.1217416

**Published:** 2024-04-04

**Authors:** Lena Rader, Siegfried Gauggel, Barbara Drueke, Lorenz Weise, Saskia Doreen Forster, Verena Mainz

**Affiliations:** Institute of Medical Psychology and Medical Sociology, University Hospital RWTH Aachen, Aachen, Germany

**Keywords:** spontaneous self-affirmation, self-esteem, structural equation modeling, psychometric properties, SSAM

## Abstract

**Introduction:**

People use coping strategies such as self-affirmation to manage threats to their self-esteem. In empirical research, self-affirmation often involves recalling personal values, strengths, or relationships to restore moral integrity. Research shows it improves attitude adjustment, resolves cognitive dissonance, and enhances well-being. Some studies stress the importance of distinguishing between different aspects of self-affirmation, like strengths or social relations. These aspects align with concepts in psychotherapy that differentiate between internal and external resource activation, benefiting health, self-esteem, and resilience. The aim of the current study was twofold: first, to independently test the three-factor structure of the Spontaneous Self-affirmation Measure (SSAM), and second, to integrate self-affirmation strategies into a broader resource activation framework as resilience factors. It also examined associations with self-esteem and effects of age, gender, and education on spontaneous self-affirmation.

**Methods:**

1,100 participants (72% female, age 18–65) were recruited online. The original three-factor structure of the SSAM (with the factors *Strengths*, *Values* and *Social relations*) was examined using structural equation modeling. Further, a theory driven two-factor structure applying an internal and external resources framework was examined, integrating the factors of the SSAM into the taxonomy of resource activation (*Internal resources: Strengths and Values; External resources: Social relations*).

**Results:**

The results of confirmatory factor analyses showed that both the original three-factor structure and the complementary two-factor structure with an *Internal resources* and *External resources* factor fit the data appropriately. All three factors of the original factor model showed a high reliability (*Strengths*: $\omega_{t}$ = 0.91, *Values*: $\omega_{t}$ = 0.91, *Social relations*: $\omega_{t}$ = 0.92). We also found measurement invariance across age, gender, and education. Furthermore, group differences regarding gender, education and ethnicity in the utilization of spontaneous self-affirmation strategies were apparent. Finally, it was demonstrated that the *Internal resources* factor of the complementary two-factor model is significantly more strongly correlated with self-esteem than the *External Resources* factor [*z* = 12.80, *p* < 0.001, 95%CIdiff (0.24, 0.33)].

**Discussion:**

The study confirms the validity of both the three-factor and two-factor structures of the SSAM. Integrating self-affirmation into the resource activation framework may facilitate applying findings from self-affirmation studies to clinical contexts.

## 1 Introduction

People generally strive to feel good about themselves (Alicke and Sedikides, 2009). In daily life, however, people often have to deal with threats to their self-esteem such as being socially rejected or negatively evaluated which can cause feelings of inadequacy (Leary, 2007). Since a threat to self-esteem challenges the motivation to feel good about oneself, this can create a cognitive dissonance (Aronson et al., 2019). Steele (1988) describes different ways of coping with cognitive dissonance, such as rationalization, external attributions of failure, or even dampening of the dissonance using alcohol or medications. All of these strategies have in common that they are meant to help one feel better and regain a sense of control. However, in a series of experiments, Steele (1988) found that when participants’ self-esteem was challenged, they sometimes did not feel the need for rationalizations or justifications. Instead, they felt better about themselves when they thought about things that mattered to them personally, like their own values. Steele (1988) called this process *self-affirmation*. Self-affirmation involves restoring one’s overall self-image through various strategies such as acts of kindness or generosity, focusing on one’s personal values or important social relationships or recalling one’s personal resources such as strengths and attributes, positive traits, skills and performances (Steele, 1988; McQueen and Klein, 2006; Harris et al., 2019). Central to this is that individuals focus on something that is meaningful to them. Although there exist various self-affirmation strategies, empirical research has mainly focused on factors such as strength, values, and social relations when studying the implementation of self-affirmation strategies. Importantly, Steele (1988) notes that self-affirmation does not resolve the dissonance induced by the self-esteem threat itself. Rather, by affirming themselves in a domain that is not affected by the threat, people can focus on the bigger picture and restore a global sense of self-worth (Steele, 1988; Aronson et al., 2019).

Several studies have demonstrated the positive effects of self-affirmation on well-being. Early experimental studies were especially interested in the effects of self-affirmation on attitude change and reduction of cognitive dissonance (Steele, 1988). In a systematic literature review, McQueen and Klein (2006) found that most studies manipulated self-affirmation by instructing participants to focus on their core values or personal strengths. Being reminded of one’s personal values for instance reduced cognitive dissonance and the need for rationalization following a forced-compliance paradigm (Aronson et al., 2019). Self-affirmation with values has also been found to improve problem-solving, reduce alcohol consumption and improve message acceptance, and to reduce cortisol levels following a laboratory stress challenge (Creswell et al., 2005, 2013; Fox et al., 2017). Studies moreover found positive effects of familial self-affirmation on different outcome measures. Reminding oneself of values that are important to one’s family members was for instance more effective in diminishing the disadvantageous influence of negative feedback than being reminded of values that are merely important to oneself (Cai et al., 2013). The study highlights the importance of distinguishing among different sources of self-affirmation (Cai et al., 2013). Similarly, Burson et al. (2012) found that focusing on self-transcendent values such as being in mutually supportive and caring relationships buffered the participants against the negative effects of social exclusion. Recently, self-affirmation interventions using different self-affirmation domains were also found to reduce depressiveness and anxiety in subclinical samples (Łakuta, 2023; Pandey et al., 2023).

After the positive effects of experimentally manipulated self-affirmation have been demonstrated in numerous studies, researchers became increasingly interested in people’s natural tendency to self-affirm when their self-image is threatened. To systematically assess people’s self-reported tendency to spontaneously self-affirm, Harris et al. (2019) developed the Spontaneous Self-affirmation Measure (SSAM). The items for the SSAM were created based on an extensive literature review of experimental self-affirmation studies. The rationale for the factorial structure of the SSAM was inferred from the types of self-affirmation interventions typically used in these studies. Harris et al. (2019) divided these studies in three categories – focusing on strengths, values, or social relations. Consequently, the authors postulated a three-factorial model with a higher-order self-affirmation factor and three first-order factors (*Strengths, Values, and Social relations*) which showed a good model fit in an initial confirmatory factor analysis [*χ*^2^(60) = 124.73, *p* < 0.001, CFI = 0.99, RMSEA = 0.05]. The authors further showed that the three-factor solution fits substantially better than a simple 1-factor solution [*χ*^2^(66) = 2139.57, *p* < 0.001]. The authors subsequently validated the SSAM in different samples where it also showed good model fit both with and without a higher-order self-esteem (RSES) and positive thinking factors [HIPT; e.g., study 2 (SSAM only): *χ*^2^(57) = 102.72, CFI = 0.97, RMSEA = 0.06]. In a series of studies, Harris et al. (2019) further found that people’s tendency to spontaneously self-affirm as assessed with the SSAM mirrors the findings of experimental studies. The SSAM has for instance been found to predict control-based optimism and systematic processing positively and optimistic denial negatively. Furthermore, the authors explored to what extent spontaneous self-affirmation can be distinguished from a general positive self-regard. This was achieved by additionally including self-esteem (Rosenberg Self-Esteem Scale, RSES) and the general attitude toward positive thinking (Habitual Index of Positive Thought, HIPT) in the factor analysis. They found strong evidence for the discriminant validity of the SSAM, as it uniquely predicted certain outcome measures (e.g., systematic processing). Importantly, the authors note that “core self-affirmation, stripped of self-esteem, appears to be associated with more systematic processing; this is consistent with assumptions underlying much experimental work using self-affirmation manipulations with health-risk information” (Harris et al., 2019, p. 25). In summary, the findings presented by Harris et al. (2019) illustrate strong support for the validity of the SSAM, and a substantial convergence between the evaluation of spontaneous self-affirmation using questionnaires and the results obtained in experimental studies involving manipulated self-affirmation.

The aim of the present study is to independently empirically validate the SSAM to allow a valid assessment of spontaneous self-affirmation. In addition to the SSAM, two other questionnaires should be mentioned here that have been used to assess people’s tendency to self-affirm: the Cognitive Self-affirmation Inclination Scale (CSAI; Pietersma and Dijkstra, 2012) and the Self-integrity scale (SIS; Sherman et al., 2009). The SIS is designed to assess feelings of moral and adaptive adequacy (e.g., “I feel that I’m basically a moral person”). The CSAI, like the SSAM, is designed to assess individual differences in self-affirmation. In the current study, we decided to focus on the SSAM for the following reasons: first, the SSAM is specifically designed to assess spontaneous self-affirmation in the face of self-esteem threats, second it assesses different self-affirmation strategies (e.g., focusing on one’s personal strengths, values or social relations), and third, it is also the most widely used questionnaire to assess spontaneous self-affirmation (e.g., Webb et al., 2020; Seaman et al., 2021; Harris et al., 2023). Since self-affirmation is a powerful resilience source, the valid measurement of the construct holds significant importance. In addition to examining the three-factor structure proposed by Harris et al. (2019), the present study aims to investigate whether the SSAM can also be integrated into a broader theoretical framework. For this, we suggest considering self-affirmation as a form of resource activation. Resource activation is used, among other things, in positive psychotherapy and strength-based approaches, involving the activation of positive emotions, character strengths, positive relationships and intrinsically motivated accomplishments (Rashid, 2015). Grawe (1997) describes resource activation as an unspecific cross-therapeutic mechanism of action in psychotherapy and its positive effects have been demonstrated in numerous studies (e.g., Goldbach et al., 2023). In the literature, resources are frequently divided into personal/individual (internal) and social (external) resources (e.g., Martin, 2002; Holmgreen et al., 2017; Salgado et al., 2022; Siegwart et al., 2022). Examples of internal resources are personal mastery beliefs (beliefs that one has the ability to control outcomes), self-efficacy beliefs, resilient coping, optimism, and a sense of coherence. Examples of external resources are social integration, emotional support from others, material support, information, advice, spiritual benefits (for religious people, e.g., going to church) social support, parental support, peer integration and school integration (National Research Council, 2003; Salgado et al., 2022; Siegwart et al., 2022). The self-affirmation factors *Strengths*, *Values*, and *Social relations* seem to fit well within this wider resource framework. Strengths and values could be classified as internal resources, while social relationships could be referred to as external resources. We suggest that self-affirmation may help to regulate self-esteem similar to recruiting internal or external resources to confirm one’s moral adequacy. The study at hand therefore also aims to explore if the internal-external resource division relates to a broader context of self-affirmation as a resilience resource and, hence, whether a complementary two-factorial structure (internal – external) also applies to the SSAM. We believe that this allows for a comparison between research on self-affirmation, predominantly conducted with healthy subjects, and investigations into resources and resource activation primarily situated within clinical contexts. This could facilitate a better translation of findings on self-affirmation into clinical settings.

To date, there are already some studies that have used the SSAM and have illustrated its areas of application and usefulness. Spontaneous self-affirmation assessed with the SSAM was for instance associated with greater happiness, hopefulness, optimism, subjective health, and personal health efficacy, as well as less anger and sadness (Emanuel et al., 2018). It also positively predicted several state well-being outcomes such as affect balance and reduced anxiety (Jessop et al., 2023). Emanuel et al. (2018) also found that people who are more exposed to discrimination experiences in their everyday life such as Black and Hispanic respondents reported engaging in more spontaneous self-affirmation which again highlights the role of spontaneous self-affirmation as a powerful resilience resource. A study by Webb et al. (2020) further found that the positive effects of spontaneous self-affirmation on self-weighing were dependent on the source of self-affirmation. Only people who had the tendency to self-affirm using their strengths were significantly less preoccupied with their weight and showed less anticipated negative affect when they weigh themselves. This effect was, however, not found for spontaneous self-affirmation using values or social relations. The results highlight the importance of the source of self-affirmation and that individual differences in the use of self-affirmation strategies influence how people deal with information that challenges their self-image (Webb et al., 2020). Study findings on spontaneous self-affirmation align well with a classification into internal and external self-affirmation strategies. For instance, a study by Harris et al. (2023) demonstrated that non-avoidant coping can be better predicted by values and strengths rather than social relations. More specifically, whereas the strengths and values factors predicted more non-avoidant coping, the social relations factor did not predict coping in any model. On a general note, the authors conclude that self-affirmation is associated with less defensive and more adaptive responses to threats (Harris et al., 2023). However, the results also indicate a more nuanced picture, with strengths and values appearing to share more common variance than with social relations. Furthermore, the findings from the initial study by Harris et al. (2019) suggest that the three self-affirmation factors both have similarities and also stand out in their connections to some outcome measures. Some findings also indicate that the *Strengths* and *Values* factors appear to be more closely related to each other than to the *Social relations* factor. For instance, *Strengths* and *Values* were both more negatively associated with avoidance denial, conscientiousness and emotional stability than *Social relations* (Harris et al., 2019).

In addition to validating the factor structure of the SSAM, the present study, like the original study by Harris et al. (2019), aims to investigate the relationship between self-affirmation and self-esteem. On a general note, self-affirmation and self-esteem are considered to be related but independent constructs (Harris et al., 2019). Self-esteem has already been described in various studies as a factor contributing to inter-individual variability in the use of self-affirmation strategies (e.g., Düring and Jessop, 2015; Harris et al., 2019). It is generally assumed that individuals with low self-esteem are less aware of their resources and therefore use less spontaneous self-affirmation (Harris et al., 2019). However, some studies also show that especially people with low self-esteem benefit from self-affirmation interventions, as these interventions bring their resources to the forefront, contributing to a more functional self-esteem regulation (e.g., less rationalization; Steele, 1988; Düring and Jessop, 2015). Düring and Jessop (2015) for instance found that trait self-esteem moderated the effect of a self-affirmation intervention (focusing on personally important values) on the participants’ openness to a health-risk message. More specifically, participants with low self-esteem showed significantly more positive attitudes, higher intentions for behavior change and less message derogation in the self-affirmation condition (writing an essay about their most important value) compared to the control condition (writing an essay about their least important value). In contrast, for participants with high self-esteem, no significant differences were found between the self-affirmation and the control condition.

There are, however, also studies that have shown the opposite effect. Zhu and Yzer (2021) recently found that participants with high self-esteem were more likely to benefit from a self-affirmation intervention (in which participants wrote a value essay or rated themselves on an attribute scale) in terms of reducing defensive responses and increasing message acceptance than participants with low self-esteem. These results were consistent across timing (self-affirmation intervention before or after health-risk message) and types of self-affirmation induction (value essay or attribute scale). The authors state that their results are consistent with the Affirmational Resources View of self-esteem which postulates that people are more resilient when facing a self-esteem threat the more self-affirmational resources they have (e.g., high self-esteem; Steele et al., 1993; Zhu and Yzer, 2021). Based on these findings, the present study hypothesizes that the Affirmational Resources View primarily applies to self-affirmation involving *Strengths* and *Values* (internal resources), but not to *Social relations* (external resources). We propose that individuals with high self-esteem are more aware of their strengths and values than those with low self-esteem, but social relationships are available as a resource to both individuals with low and high self-esteem.

Altogether, the aim of the study was on the one hand to validate the original 3-factorial structure of the SSAM and on the other hand to investigate an additional 2-factorial structure with an *Internal* and *External resources* factor. The 2-factorial structure could be of interest for researchers who want to investigate spontaneous self-affirmation in the sense of resource activation. In this context, we also aim to investigate whether the *Internal resources* factor is more strongly associated with self-esteem than the *External resources* factor, which could provide more nuanced insights into the Affirmational Resources View. Moreover, the present study aims to assess the psychometric properties of the SSAM such as the reliability of the self-affirmation factors as well as the measurement invariance across age, gender, and education. Finally, given a large sample, the study could reflect on group differences in spontaneous self-affirmation.

## 2 Methods

### 2.1 Participants

In total, 1,100 participants were recruited, whereby 550 were sampled from the United Kingdom and 550 were sampled from the US. 790 participants identified as female, 307 as male, and 3 as non-binary. Participants age ranged from 18 to 65 years (*M* = 47, *SD* = 12). 51% of the participants had a high school degree, 41% a university degree, 2% a middle school degree, and 6% indicated having another degree. The most commonly reported nationalities, respectively ethnicities were British (34.36%), followed by American (22.09%), White (12.27%), Black (2.18%) and Asian (1.45%).^1^ The sociodemographic characteristics of the sample can also be found in Table 1. This study was reviewed and approved by the local Ethics Committee (EK 345/21). The study was conducted as part of a larger research project which was pre-registered (https://doi.org/10.23668/psycharchives.5105). Please note, however, that the exact analyses conducted in the current study were not outlined in detail in the pre-registration.

**Table 1:** Sociodemographic characteristics of the sample used in the current study and of the samples in Harris et al.’s (2019) preliminary studies.

	Current study					Harris et al. (2019)		
						Preliminary study 1	Preliminary study 2	
		United Kingdom (N)	United States (N)	Total (N)			Sample 1	Sample 2
Age (years)	18–34	119	83	202	*M* = 47, SD = 12	*M* = 21.2, SD = 5.8	*M* = 20.0, SD = 1.3	*M* = 20.1, SD = 1.2
	35–49	162	204	366				
	50–65	269	263	532				
Gender	Female	366	424	790	72%	66%	85%	91%
	Male	182	125	307				
	Non-binary	2	1	3				
Education	Middle school	17	4	21		Students	Students	Students
	High school	285	277	562				
	University	219	228	447				
		550	550	1,100		850	95	84

### 2.2 Material

#### 2.2.1 Self-affirmation

The Spontaneous Self-Affirmation Measure (SSAM) by Harris et al. (2019) is a 13-item self-report measure that is comprised of a general higher-order self-affirmation factor (comparable to the g-factor in intelligence research) and three first-order factors *Values* (item 2, 3, 5 and 12), *Strengths* (item 1, 8, 9 and 13), and *Social relations* (item 4, 6, 7, 10 and 11). Items are answered on a scale from 1 (“completely disagree”) to 7 (“completely agree”). A high score thus indicates a high degree of self-affirmation.

#### 2.2.2 Self-esteem

The Rosenberg Self-esteem scale (RSES; Rosenberg, 1965) is a 10-item self-report measure that can be used to assess self-esteem. The items are answered on a scale ranging from 1 (“strongly agree”) to 4 (“strongly disagree”). Item 1, 3, 4, 7 and 10 were reverse-coded so that a high score on the RSES indicates a high self-esteem.

### 2.3 Procedure

#### 2.3.1 Data collection

Participants were recruited via the online platform Qualtrics (Qualtrics Labs Inc., Provo, Utah).^2^ They were first asked to answer sociodemographic questions on age, gender, native language, nationality, and level of education (school degree). Participants were excluded if they did not meet the following criteria: age between 18 and 65 years, fluent in English. They were further excluded if they did not pass the data integrity check (see below). The participants then answered the SSAM (Harris et al., 2019) and the Rosenberg self-esteem scale (Rosenberg, 1965) in randomized order.

#### 2.3.2 Data integrity check

Data were screened for straight-liners (participants who gave the same response on all items) during data collection. Straight-liners were defined as participants with a variance of zero across all items of a questionnaire (SSAM or RSES). No participants had to be excluded based on this criterion.

### 2.4 Data analysis

#### 2.4.1 Confirmatory factor analysis of the SSAM

**Figure 1:**
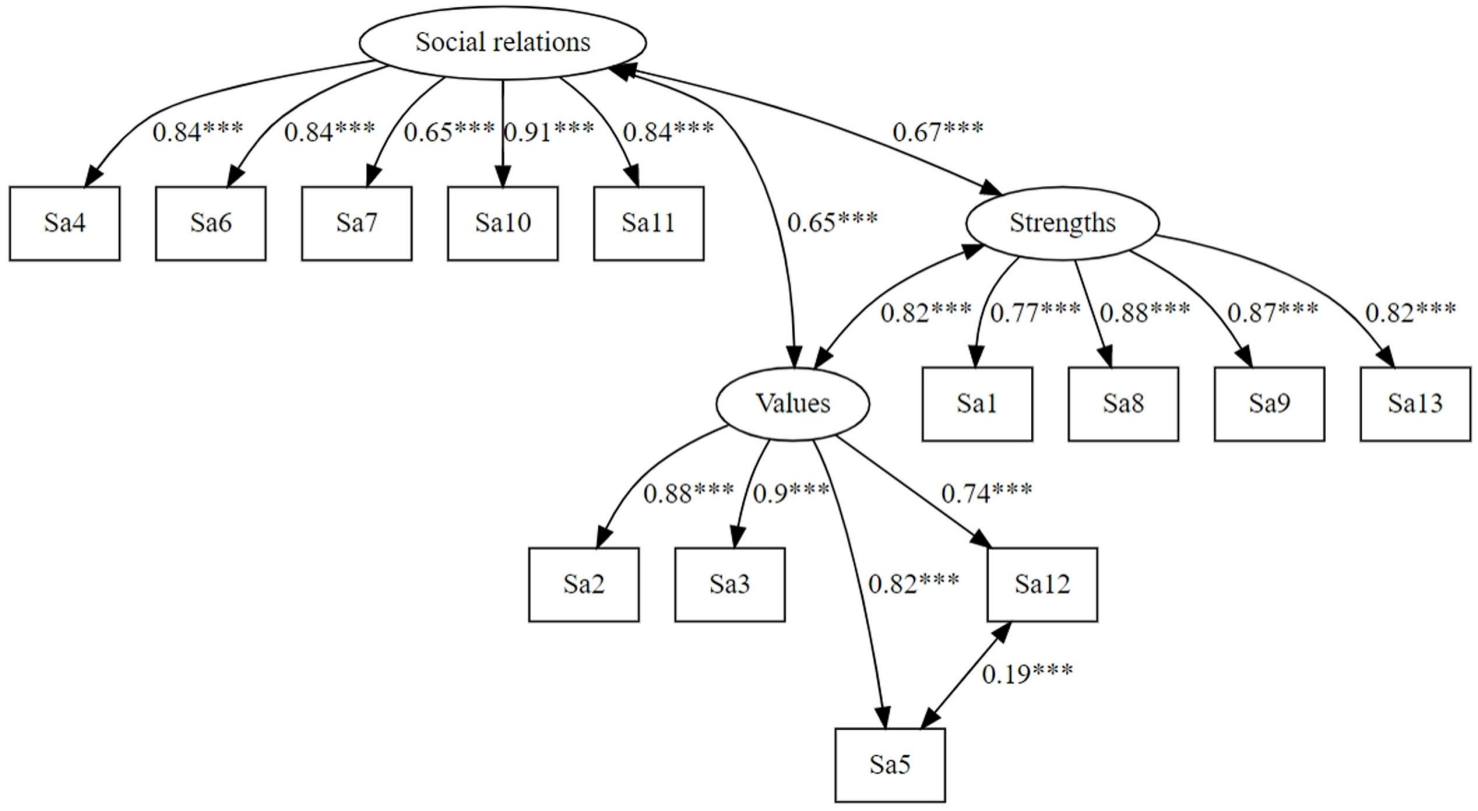
Original 3-factor model of the SSAM proposed by Harris et al. (2019) with three first-order factors (Social relations, Values, Strengths) and a correlated residual between item 5 and 12.

**Figure 2:**
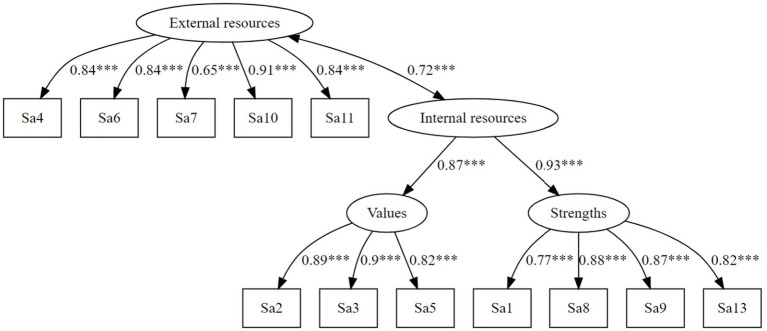
Alternative 2-factor model of the SSAM proposed in the current with an internal resources and an external resources factor.

First, the fit of the original factorial structure of the SSAM found by Harris et al. (2019) was examined in a confirmatory factor analysis (CFA). As in the original article, the model was comprised of a higher-order self-affirmation factor and three first-order factors (*Values*, *Strengths*, *Social relations*) and a correlated residual between item 5 and 12. To distinguish self-affirmation from positive self-regard, self-esteem was also included in the model (the model can be found in Supplementary Figure S1).^3^ Next, the 3-factor model was tested without self-esteem (see Figure 1). Moreover, the fit of an additional 2-factor model for the SSAM was examined. For this purpose, the items of the SSAM were assigned to two factors: the *Social relations* items were assigned to an *External resources* factor, and the factors *Strengths* and *Values* were treated as first-order factors under a higher-order factor labeled *Internal resources* (see Figure 2). This complementary model was examined to assess whether self-affirmation and specifically the SSAM can also be integrated into a resource activation framework.

Prior to testing the models, multivariate normality was assessed using the Mardia test (Korkmaz et al., 2014). Since the critical ratio of multivariate kurtosis was above 0.5, the assumption of multivariate normality was violated (Byrne, 2010) and robust maximum likelihood with a mean and variance-adjusted Chi-square test was chosen as estimation method (Maydeu-Olivares, 2017). The appropriateness of the CFA models was assessed using measures of global and incremental fit. The *χ*^2^ likelihood ratio and the Root Mean Square Error of Approximation (RMSEA), which estimate the discrepancy between the estimated model and the empirical data, were used to assess the global model fit. A non-significant *p*-value of the *χ*^2^-test indicates that the hypothesized model fits the empirical data appropriately. However, because the *χ*^2^-test statistic has been shown to be sensitive to large sample sizes (Kline, 2011), the *χ*^2^/degrees of freedom (*df*) ratio will be additionally reported. A *χ*^2^/*df* ratio smaller than 2.5 and a RMSEA <0.06 are generally considered to indicate acceptable model fit (Hu and Bentler, 1999; Jackson et al., 2009). In addition, the Standardized Root Mean Square Residual (SRMR) will be reported, since it has been shown to be robust to the method used to estimate model parameters (Shi and Maydeu-Olivares, 2020). SRMR values <0.08 indicate acceptable model-data fit (Hu and Bentler, 1999). The Comparative Fit Index (CFI) was used to assess the incremental model fit. The CFI indicates increases in the model fit of the proposed model relative to a null model. A CFI value >0.95 indicates a good model fit (Hu and Bentler, 1999; Jackson et al., 2009). Note that scaled CFI (${\text{CFI}}_{s}$) and RMSEA (${\text{RMSEA}}_{s}$) based on the mean and variance-adjusted Chi-square test will be reported. Based on a simulation study, Golino et al. (2020) advised to interpret CFA model fit indices as relative rather than absolute indices. The model fit indices used in the current study will thus be interpreted as relative rather than absolute. The model with the best overall fit to the data compared to the other models will be preferred. We also used the Akaike Information Criterion (AIC) to assess the relative fit of different models of the SSAM. The AIC indicates the goodness of fit based on the maximum likelihood value while taking into account the number of parameters estimated in the model. Lower AIC values indicate better model fit. As a rule of thumb, it has been suggested that a model is considered empirically poor if the AIC of the model is more than 10 units greater than the lowest AIC (Cavanaugh and Neath, 2019).

#### 2.4.2 Reliability of the SSAM

The reliability was estimated using McDonald’s Omega (ω; Graham, 2006; Bühner, 2011). The reliability of the three first-order factors *Strengths*, *Values* and *Social relations* (resp. *External resources* in the 2-factor model) was estimated using total omega ($\omega_{t}$). The reliability of the higher-order factor *Internal resources* of the 2-factor SSAM model was estimated using hierarchical omega ($\omega_{h}$; Savalei et al., 2019; Flora, 2020). In order to select an appropriate reliability coefficient, a confirmatory factor analysis was conducted beforehand to determine which measurement model applies to the SSAM. A detailed description of testing measurement models of classical test theory using structural equation modeling can for instance be found in Bühner (2011) and Graham (2006). The measurement models were estimated for the three first-order factors *Strengths*, *Values* and *Social relations* (resp. *External resources*) of the SSAM. Robust maximum likelihood with mean and variance-adjusted Chi-test were used as estimation method. The same cut-offs for model fit indices as in the confirmatory factor analysis described above were used. The results of this analysis can be found in the Supplementary Table S1.

#### 2.4.3 Associations between the SSAM factors and self-esteem

To examine whether the Affirmational Resources View might be better explained by internal resources (strengths and values) than by external resources (social relations), the associations between the self-affirmation factors *Internal resources* and *External resources* of the 2-factor SSAM model and self-esteem were assessed using Pearson’s r and tested for statistical significance using the Fisher’s z test and the 95% confidence interval of the difference between the two correlations (95% CI*diff*; Diedenhofen and Musch, 2015). Statistical significance of the difference in the correlations was assumed when the *p*-value of the z-test was <0.05.

#### 2.4.4 Measurement invariance of the SSAM across age, gender and education

To better understand the alternative two-factor structure of the SSAM (with an *Internal resources* and *External resources* factor) and further examine its validity, measurement invariance was assessed across different sociodemographic groups. To assess measurement invariance across age, participants were divided into three age groups: 18–34 years (*n* = 202), 35–49 years (*n* = 366), and 50–65 years (*n* = 532). Regarding education, only participants with a high school (*n* = 562) and university degree (*n* = 447) could be compared, since the number of participants with middle school degree (*n* = 11) was too small. Also, only participants who indicated being female (*n* = 790) or male (*n* = 307) were included in this analysis since only three participants indicated being non-binary. Differences in CFI_s_ ($\Delta {\text{CFI}}_{s}$) between the different models were inspected to assess whether the null hypothesis of measurement invariance should be rejected. Based on a simulation study, Cheung and Rensvold (2002) proposed that measurement invariance can be assumed if $\Delta CFI_{s}$ is smaller than or equal to −0.01.

#### 2.4.5 Effect of age, gender, education and ethnicity on SSAM means

After ensuring the measurement invariance of the SSAM, the influence of various sociodemographic variables such as age, gender, education, and ethnicity on spontaneous self-affirmation was examined. Based on a national survey conducted by Emanuel et al. (2018) in the United States, higher spontaneous self-affirmation was expected in older participants and Black participants (*n* = 26) compared to White (*n* = 143) and Asian (*n* = 14) participants.^4^ No differences in spontaneous self-affirmation regarding gender and education were expected. In advance of this analysis, the assumption of homoscedasticity was checked for each group comparison using a Levene’s test, which is more robust against violations of normality than other traditional tests (Beyene and Bekele, 2016). A *p*-value less than 0.05 for the Levene statistic indicates that the assumption of equal variances among the groups should be rejected. If the assumption of equal variances was met, a one-way analysis of variance (ANOVA) was conducted. If the assumption was not met (which was only the case for the comparison of the *Internal resources* means between participants with high school and university degree), the group comparison was performed using a non-parametric Kruskal-Wallis test (Hecke, 2012). Three ANOVAs were conducted for each sociodemographic variable (e.g., age). In each analysis, the means across all items of the *Internal resources* factor, the *External resources* factor, and across all SSAM items were entered as the dependent variable. The sociodemographic variables–age, gender, education, and ethnicity–were defined as independent variables. We used the same groups for age, gender, and education in this investigation as those used to study measurement invariance (e.g., age groups 18–34 years, 35–49 years and 60–65 years). In case more than two groups were compared, post-hoc tests were conducted using Tukey HSD. Effect sizes for the ANOVAs will be reported using Cohen’s f. Effect sizes *f* = 0.10 are considered small, *f* = 0.25 medium and *f* = 0.40 large (Kang, 2021). In case the Kruskal-Wallis test was used, η^2^ will be reported as effect size. A post-hoc power analysis was conducted for each ANOVA using G*Power (Version 3.1.9.4). The results of the Levene’s test and the post-hoc power analyses can be found in Supplementary Tables S2, S3, respectively.

All data and the code behind these analyses have been made publicly available at the Open Science Framework and can be accessed at https://osf.io/62c3r/.

## 3 Results

### 3.1 Confirmatory factor analysis of the SSAM

The original model with two higher-order factors (*Self-esteem, Self-affirmation*) and three first-order self-affirmation factors (*Strengths, Values, Social relations*) fit the data appropriately according to absolute fit indices but not according to incremental fit indices [*χ*^2^(225) = 923.78, *p* < 0.001, *χ*^2^/*df* = 4.11, CFI_s_ = 0.747, RMSEA_s_ = 0.053, SRMR = 0.062, AIC = 63,645.09]. The original model with only three first-order factors (shown in Figure 1) showed an appropriate fit [*χ*^2^(61) = 353.64, *p* < 0.001, *χ*^2^/*df =* 5.79, CFI_s_ = 0.832, RMSEA_s_ = 0.066, SRMR = 0.051, AIC = 42,981.15]. The alternative 2-factor model (shown in Figure 2) also showed an appropriate model fit *χ*^2^(51) = 275.50, p < 0.001, *χ*^2^/*df* = 5.40, CFI_s_ = 0.869, RMSEA_s_ = 0.063, SRMR = 0.042, AIC = 39,798.72. Table 2 shows the model fit indices of the original 3-factor and complementary 2-factor models of the SSAM.

**Table 2:** Model fit indices of the 3-factor (original model) and 2-factor (alternative model) SSAM models.

Model	*χ* ^2^	*df*	*χ*^2^/*df*	CFI_s_	RMSEA_s_	SRMR	AIC
Original model (incl. Self-esteem)	923.78*	225	4.11	0.747	0.053	0.062	63645.09
Original model	353.64*	61	5.79	0.832	0.066	0.051	42981.15
Two-factor model	275.50	51	5.40	0.869	0.063	0.042	39798.72

Original model (incl. Self-esteem) = 2 higher-order factors (Self-esteem, Self-affirmation), 3 first-order self-affirmation factors (Strengths, Values, Social relations), correlated residual between item 5 and 12 (Supplementary Figure S1); Original model, Model shown in Figure 1; Two-factor model, Model shown in Figure 2; CFI_s_, scaled Comparative Fit Index; RMSEA_s_, scaled Root Mean Square Error of Approximation; SRMR, Standardized Root Mean Square Residual; AIC, Akaike Information Criterion, *p < 0.001.

### 3.2 Reliability of the SSAM

Since the tau-equivalent model had to be rejected for all three first-order factors (*Strengths*, *Values* and *Social relations*, resp. *External resources*) and the essentially tau-equivalent model could not be estimated, the tau-congeneric model fit the data best (see Supplementary material S2) and McDonald’s Omega (ω) was chosen as an appropriate estimation method to determine the reliability of the SSAM. All three first-order factors showed a high reliability (*Social relations/External resources:* $\omega_{t}$ = 0.92; *Strengths*: $\omega_{t}$ = 0.91, *Values*: $\omega_{t}$ = 0.91). The higher-order factor *Internal resources* also showed a high reliability ($\omega_{h}$= 0.82).

### 3.3 Associations between the SSAM factors and self-esteem

**Figure 3:**
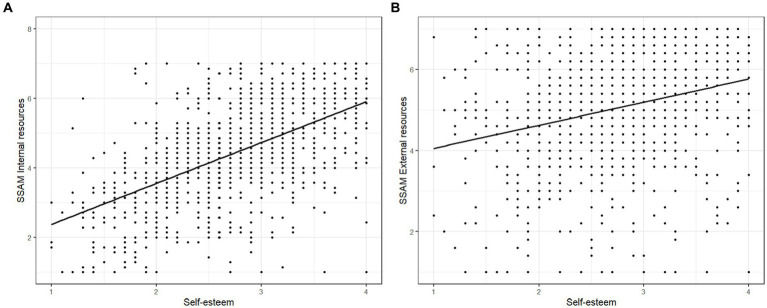
Associations between the internal resources **(A)** and external resources **(B)** factors of the 2-factor SSAM model and self-esteem.

Next, the associations between the *Internal resources* and *External resources* factors of the 2-factor SSAM model and self-esteem were assessed. Although both factors were significantly associated with self-esteem [*Internal resources*: *r* = 0.55, *t* (1098) = 22.22, *p* < 0.001; *External resources*: *r* = 0.27, *t* (1098) = 9.35, *p* < 0.001], the association between the *Internal resources* factor and self-esteem was significantly higher [*z* = 12.80, *p* < 0.001, 95%CI_diff_ (0.24, 0.33)]. Figure 3 shows the scatterplots on the association between the *Internal resources* (A) and *External resources* (B) factors of the SSAM and self-esteem. In addition, the associations between the two fist-order factors *Strengths* and *Values* and self-esteem were assessed. The *Strengths* factor [*r* = 0.56, *t* (1098) = 22.75, *p* < 0.001] was significantly higher associated with self-esteem than the *Values* factor [*r* = 0.46, *t* (1098) = 17.25, *p* < 0.001; *z* = 5.82, *p* < 0.001, 95%CIiff (0.069, 0.139)].

### 3.4 Measurement invariance of the SSAM across age, gender and education

Next, measurement invariance of the 2-factor SSAM model (shown in Figure 2) was assessed across age, gender, and education. For all three variables, measurement invariance was achieved at scalar level (see Table 3). Hence, no significant deterioration of the model fit (based on $\Delta CFI_{s})$ was found when the factor loadings and item intercepts were constrained to be equal for female and male participants, respectively for participants of the three age groups, or participants with high school vs. university degree. The factor loadings and item intercepts could thus be shown to be invariant across age, gender and education. The CFI_s_ and ΔCFI_s_ of the different models can be found in Table 3.

**Table 3:** Measurement invariance of the complementary 2-factor model of the SSAM across age, gender and education.

Group	Level of measurement invariance	*χ* ^2^	*df*	*p*	CFI_s_	ΔCFI_s_
Age	Configural	286.06	150	<0.001	0.919	
	Metric	316.85	170	<0.001	0.913	−0.006
	Scalar	337.54	186	<0.001	0.910	−0.003
Gender	Configural	545.38	100	<0.001	0.958	
	Metric	557.11	110	<0.001	0.958	0
	Scalar	588.44	118	<0.001	0.956	−0.002
Education	Configural	229.90	100	<0.001	0.912	
	Metric	243.93	110	<0.001	0.909	−0.003
	Scalar	262.23	118	<0.001	0.902	−0.007

Configural, Measurement invariance while allowing item loadings and intercepts to vary per group; Metric, Measurement invariance at the level of fixed item loadings for the groups tested; Scalar, Measurement invariance at the level of fixed item loadings and intercepts for the groups tested; df, degrees of freedom; CFI_s_, scaled Comparative Fit Index; ΔCFI_s_, Difference in scaled Comparative Fit Index compared to the more relaxed model.

### 3.5 Effect of age, gender, education and ethnicity on SSAM means

The ANOVA revealed no significant differences between the three age groups in the SSAM means (see Table 4). Regarding gender, the ANOVA revealed a significant difference between female and male participants, both on the *Internal resources* [*F* (1, 1,095) = 6.58, *p* = 0.010, *f* = 0.08] and *External resources* [*F* (1, 1,095) = 5.86, *p* = 0.016, *f* = 0.07] factors of the SSAM. The overall SSAM mean did not significantly differ between females and males [*F* (1, 1,095) = 0.030, *p* = 0.587]. For education, a significant difference between participants with high school and university degree was only found on the *Internal resources* factor [*χ*^2^(1) = 9.18, *p* = 0.002, η^2^ = 0.01]. Lastly, significant differences between Black, White and Asian participants were found on the *Internal resources* factor [*F* (2,180) = 9.73, *p* < 0.001, *f* = 0.33] and the overall SSAM mean [*F* (2,180) = 6.11, *p* = 0.002, *f* = 0.36]. Regarding the *Internal resources* factor, *post hoc* comparisons using the Tukey HSD test indicated that the mean score of the Black participants (*M* = 5.59, *SD* = 0.97) was significantly higher than that of the White participants (*M* = 4.43, *SD* = 1.34). The same pattern emerged for the overall SSAM mean with Black participants (*M* = 5.62, *SD* = 0.98) having a significantly higher mean than White participants (*M* = 4.79, *SD* = 1.17). Table 4 additionally shows the means and standard deviations of the Rosenberg Self-Esteem Scale (RSES) across the different sociodemographic groups. As a general trend, it can be seen that participants with higher means on the *Internal resources* SSAM factor also show higher self-esteem means which is in line with the positive association found between the *Internal resources* factor and self-esteem. Supplementary Table S3 shows the results and parameters of the power analyses for each ANOVAs.

**Table 4:** Means and standard deviations of the spontaneous self-affirmation measure and the Rosenberg self-esteem scale as well F-statistic and Cohen’s f effect size of the ANOVAs with the SSAM factors across age, gender, education and ethnicity.

Sociodemographic groups		Internal SSAM factor			External SSAM factor			Overall SSAM			Self-esteem	
		*M*	SD	*F* (f)	*M*	SD	*F* (f)	*M*	SD	*F* (f)	*M*	SD
Age	18–34	4.37	1.45	1.24 (0.03)	5.11	1.34	0.26 (0.02)	4.68	1.29	0.24 (0.01)	2.57	0.64
	35–49	4.40	1.37		5.03	1.29		4.66	1.23		2.71	0.64
	50–65	4.48	1.30		5.04	1.37		4.72	1.20		2.83	0.61
Gender	Female	4.37	1.32	6.58* (0.08)	5.11	1.32	5.86* (0.07)	4.68	1.22	0.29 (0.02)	2.69	0.63
	Male	4.61	1.33		4.89	1.37		4.72	1.24		2.91	0.60
Education	High school	4.33	1.39	9.18^1^* (0.01)^2^	5.08	1.35	0.73 (0.03)	4.64	1.25	1.86 (0.04)	2.67	0.66
	University	4.56	1.27		5.00	1.27		4.75	1.17		2.85	0.59
Ethnicity	Black	5.59	0.97	9.73*** (0.33)	5.65	1.15	1.01 (0.11)	5.62	0.98	6.11** (0.26)	3.05	0.64
	White	4.43	1.34		5.29	1.20		4.79	1.17		2.62	0.65
	Asian	5.16	1.54		5.50	1.70		5.30	1.55		2.86	0.71

F, F-statistic of one-way ANOVA; f, Cohen’s d effect size of one-way ANOVA; M, Mean; SD, Standard deviation; SSAM, Spontaneous Self-Affirmation Measure; ^1^since the Levene’s test was significant, since analysis was conducted using the non-parametric Kruskal-Wallis test; ^2^η^2^ is reported as effect size for the Kruskal-Wallis test; Please note the grouping of the participants into ethnic groups was only possible because some participants identified their ethnic background as their nationality; *p < 0.05; **p < 0.01; ***p < 0.001.

## 4 Discussion

Self-affirmation has proven to be a helpful strategy in numerous studies for developing an adaptive response to criticism or rejection, thereby promoting the potential for personal growth (e.g., Harris et al., 2019). The SSAM is currently the only questionnaire specifically designed to assess spontaneous self-affirmation, and its utility and predictive validity have already been demonstrated in a number of studies (Emanuel et al., 2018; Webb et al., 2020; Harris et al., 2023; Jessop et al., 2023). The current study examined the factor structure and the psychometric properties of the SSAM in a large sample (*N* = 1,100). First, we assessed the fit of the original 3-factor model by Harris et al. (2019) which was based on experimental self-affirmation manipulations (focusing on strengths, values or social relations). The original three-factor model was once tested with and without a self-esteem factor to more closely match the goal of a replication of the original study by Harris et al. (2019). In addition, we assessed the fit a complementary 2-factor model in which self-affirmation is viewed as a form of resource activation. With the resource activation view in mind, it was assessed whether the three SSAM factors can be classified into internal resources (*Values* and *Strengths*) and external resources (*Social relations*). The results of the current study underline the validity of the SSAM as the 3-factor model showed an acceptable fit to the data. Moreover, all three factors first-order factors (*Strengths*, *Values* and *Social relations*, resp. *External resources* in the 2-factor model) showed a high reliability. The higher-order factors *Internal resources* of the 2-factor model also showed a high reliability. At this point, we would like to emphasize once again that the aim of the study was not to present an alternative but rather a complement to the 3-factor structure of the SSAM. There are also reasons to prefer the 3-factor structure over a 2-factor structure. As mentioned earlier, self-affirmation factors show individual associations with different outcome measures (see Harris et al., 2019). At this juncture, the association between the *Strengths* factor of the SSAM and self-esteem should be highlighted. Harris et al. (2019) demonstrated that the *Strengths* factor negatively predicted self-clarity and was the only SSAM factor to predict defensiveness. In contrast, for example, the *Values* factor showed a positive association with self-clarity. It becomes clear, therefore, that the *Strengths* factor can partially reflect characteristics that are atypical of self-affirmation, which are more commonly found in strategies such as self-enhancement (see, for example, Hepper et al., 2010). Ultimately, the choice of factor structure for analysis likely depends on the research question. If one is interested in exploring the relationships between different self-affirmation strategies and outcome measures, for example, to examine their adaptivity vs. maladaptivity, the 3-factor structure may be more suitable. For researchers who wish to investigate differences in the use of self-affirmation among individuals with high and low self-esteem and consider self-affirmation in terms of resource activation, the two-factor structure could be helpful as a complementary approach. In the following sections, we would like to outline some of the implications that, in our view, arise from the two-factor structure of the SSAM.

While there is substantial evidence supporting the positive effects of self-affirmation, there are also instances in which some of the participants did not benefit from the interventions (e.g., Zhu and Yzer, 2021) and it is therefore of great importance to further understand individual differences in (spontaneous) self-affirmation. The “Trigger and Channel” framework, proposed by Ferrer and Cohen (2019), is a theoretical model that aims to explain when self-affirmation interventions are most effective and postulates three conditions that have to be met: (1) the presence or perception of a threat (to the self, one’s health, etc.), (2) the availability of resources (e.g., infrastructure, social support) and (3) the temporal proximity of the threat and self-affirmation. According to the authors, positive behavioral change is most likely when a person perceives a threat to their self-image (e.g., smokers being informed about the health risks of smoking) and resources are available to trigger motivation for change, which can then be channeled into actual behavior which may eventually foster a positive upward spiral (Ferrer and Cohen, 2019). One of the crucial points in this model is the availability of resources that enable self-affirmation when a threat to the self is present. At this juncture, the previously described Affirmational Resources View, can be invoked to understand why people may differ in their access to their resources. It is assumed that people with high self-esteem have greater awareness of their resources and better access to them (Harris et al., 2019). Following this logic, individuals with high self-esteem can employ more self-affirmation strategies and, as a result, may rely less on other defensive strategies such as self-protection (e.g., rationalizations, attribution bias) to cope with criticism or rejection (Cohen and Sherman, 2014; Harris et al., 2019). The study by Zhu and Yzer (2021) for instance showed that participants with high self-esteem benefited more from a self-affirmation intervention that focused on strengths and values (which we classified as internal resources) compared to participants with low self-esteem. We believe that this is an important finding because individuals with low self-esteem should, in theory, benefit more from such interventions. This is significant because low self-esteem is associated with dysfunctional self-esteem regulation strategies like self-protection (Hepper et al., 2010), and low self-esteem is also described as a risk factor for the development of mental disorders such as depression (e.g., Zhou et al., 2020). Therefore, research should focus both on understanding the utilization of self-affirmation strategies in individuals with low self-esteem, as well as on investigating how this utilization can be promoted. In the present study, it was also demonstrated that the *Internal resources* factor is significantly more strongly associated with self-esteem than the *External resources* factor which aligns with the findings of Zhu and Yzer (2021). This could potentially be attributed to the fact that individuals with high self-esteem may have better access to their internal resources, such as strength and values, compared to individuals with low self-esteem, while external resources may be similarly accessible to both high and low self-esteem individuals.

If one remains within the resource activation framework, the knowledge of one’s internal resources (one’s strengths and values) versus the knowledge of external resources (friends, family etc.) strongly resembles the distinction between self-knowledge and other-knowledge. Several studies have already demonstrated that knowledge about oneself (self-knowledge) can be recalled more effectively than knowledge about others (Rogers et al. 1977; Symons and Johnson, 1997). This is explained, in part, by the fact that these pieces of information are retrieved from different networks. Nakao et al. (2009) for instance state that „self-knowledge, which is knowledge of one’s own personal traits and abilities, is a unique cognitive structure “(page 69). A study by Chen et al. (2021) has shown that the core self-network (mainly the ACC) and the salience network (mainly the insula) are specifically involved in the retrieval of self-knowledge as distinct from knowledge about close others. It appears, therefore, that self-knowledge is generally easier to access than knowledge about close others. It might therefore be speculated, that internal resources (relying on self-knowledge) are used more frequently to self-affirm than external resources (relying on other-knowledge). However, we further argue that this is not the case for everyone and that self-esteem plays a crucial role in the effective retrieval of internal resources. This assumption is based on studies that have found a connection between self-esteem and information processing. For example, a study by Vandellen et al. (2012) demonstrated that individuals with low self-esteem were more focused on their environment and social cues after experiencing rejection, as compared to those with high self-esteem. This could explain why individuals with low self-esteem may have difficulty accessing their internal resources when facing rejection or criticism, and therefore, may use spontaneous self-affirmation less often to regulate their self-esteem. This aligns with studies showing that individuals with low self-esteem tend to resort to defensive strategies like self-protection, which do not require access to self-relevant information (Hepper et al., 2010). In summary, there is evidence suggesting that people can access knowledge about themselves (internal resources) more readily than knowledge about others (external resources), as reflected in neuroimaging studies that reveal the activation of different networks. Simultaneously, there are inter-individual differences in the availability of these resources that can partly be explained by variations in self-esteem, given its impact on information processing. Consequently, and in our view, the crucial point is that internal resources are more challenging to access for individuals with low self-esteem. This is also reflected in the results of the current study, as a significantly stronger correlation was found between self-esteem and *Internal resources* compared to self-esteem and *External resources*. With regard to the Affirmational Resource View, our argument is that internal resources, such as one’s own strengths and values, are more accessible to individuals with high self-esteem, while external resources are less dependent on self-esteem. This increased accessibility of internal resources might explain the greater use of spontaneous self-affirmation strategies and, consequently, a higher level of resilience against self-threats for people with higher self-esteem compared to people with lower self-esteem. Future studies should therefore investigate how to promote positive self-focused attention in individuals with low self-esteem to facilitate the retrieval of internal resources and, consequently, encourage the use of spontaneous self-affirmation.

The current study was also the first to assess the measurement invariance of the SSAM. We found measurement invariance at scalar level (fixing item loadings and intercepts) across age, gender and education. Measurement invariance at scalar level is a requirement for the interpretation of latent means (Cheung and Rensvold, 2002). Since this requirement was met, we aimed to further understand the complementary 2-factor model of the SSAM and compared the means of the *Internal resources* and *External resources* factor across different sociodemographic groups in a next step. Contrary to our predictions, we did not find a significant effect of age. Only on the *Internal resources* factor we found a (non-significant) trend of increasing self-affirmation means with age in line with the findings by Emanuel et al. (2018). Also against our predictions, we found significant effects of gender and education on self-affirmation. Interestingly, female participants had significantly higher means on the *External resources* factor while male participants had significantly higher means on the *Internal resources* factor. Female participants thus seemed to rely more on their social environment to self-affirm while male participants seemed to rely more on their strengths and values. Regarding the effect of the participants’ ethnicity on self-affirmation we found significant group differences on the *Internal resources* factor and on the overall SSAM score. Post-hoc tests revealed that Black participants had significantly higher means on the *Internal resources* factor, followed by Asian and White participants (the SSAM means of the different groups are also shown in Table 2). This pattern was also found on the *External resources* factor but the mean differences were not significant. These results are largely in line with the findings by Emanuel et al. (2018). Participants who are potentially more frequently exposed to discrimination experiences in their daily life showed higher self-affirmation scores, although it has to be noted that such data was not assessed in the current study and needs further investigation. Interestingly, Black and Asian participants also showed higher self-esteem means compared to White participants (see Table 2). Here, it should be noted, however, that the results regarding ethnicity should be interpreted with caution, as the groups of Black and Asian participants were extremely small. Post-hoc power analyses, based on effect sizes, revealed that the ANOVAs for age, gender, and education were underpowered, while a high power was calculated for ethnicity (see Supplementary Table S3). Therefore, the results of the present study should be validated using a larger sample. A question left unresolved by the present study concerns the direction of this effect: Does frequent self-affirmation usage result in higher self-esteem, or does greater self-esteem facilitate the adoption of more self-affirmation strategies?

A longitudinal study by Lannin et al. (2021) contributed to a better understanding of the directionality of this influence. The results of their study revealed that higher self-affirmation scores at time point 1 led to higher self-esteem scores at time point 2, but the reverse effect was not statistically significant. For the current study, this could potentially imply that individuals who experience more everyday discrimination may use more self-affirmation strategies to regulate their self-esteem, which could ultimately result in higher self-esteem over the long term. It would be interesting to additionally investigate whether Black or Asian participants, through increased use of self-affirmation, concurrently employ fewer defensive strategies such as self-protection. Tesser et al. (2000) found in a study that people tend to be self-esteem satisfiers rather than maximizers. This means that when individuals possess a well-functioning self-esteem regulation strategy (e.g., self-affirmation), there may be no need to resort to additional strategies (e.g., self-protection) to enhance or regulate their self-esteem. Furthermore, it would be intriguing to understand what enables Black and Asian participants to utilize more self-affirmation strategies. This could contribute to a better understanding of inter-individual differences in the use of self-affirmation strategies. These considerations, however, are currently speculative and should be examined, for example, through longitudinal study designs. Lastly, it is worth noting that the results of the current study suggest that self-affirmation can be helpful in coping with stereotype threat which is defined as the awareness that one might be negatively evaluated based on one’s social identity (e.g., gender, ethnicity, social class.; Steele, 1997). The positive effects of self-affirmation on stereotype threat have so far mainly been investigated in the context of gender (e.g., Martens et al., 2006; Voisin et al., 2019) and ethnicity (e.g., Adams et al., 2006; Cohen et al., 2006; Cohen and Sherman, 2014) and should also be extended to other groups that may be affected by stereotype threat, such as the LGBTQAI+ community.

Some limitations of the current study should be noted. First, participants were recruited via an online platform which can compromise data integrity. However, based on the data integrity checks it can at least be ruled out that participants gave the same answer on all items of the SSAM or RSES as sufficient variance in the data was found. Moreover, due to the online recruitment method, contextual factors such as being in a noisy environment were not assessed which might have biased the results of the current study. Regarding the characteristics of the sample it should be noted that people with lower educational attainment were rather underrepresented as there were comparably few participants with middle school degree compared to high school and university degree. As outlined above the grouping of the participants into ethnic groups was only possible because some participants identified their ethnic background as their nationality. Therefore, the categorization is artificial, and the results should be interpreted with caution. Another limitation to note is that the groups were of varying sizes, with the group of Asian participants being particularly small. Bearing the limitations in mind, still, the results are largely consistent with previous studies and can have significant implications for the application of self-affirmation interventions in ethnic minority groups.

The complementary two-factorial structure of the SSAM proposed in the current study should be validated in future studies as the distinction between internal and external self-affirmation resources might help to inform and personalize self-affirmation interventions. The distinct correlations between the *Internal resources* and *External resources* factor and self-esteem could contribute to understanding why certain studies suggest that not all self-affirmation interventions yield equal effectiveness for every individual. Since people with low self-esteem report using their internal resources less frequently in the current study it is all the more important to identify factors that could amplify the access to internal resources. Future (preferably experimental) studies should, therefore, validate whether self-affirmation interventions with internal and external resources function differently for individuals with high and low self-esteem. Additionally, they should investigate the factors contributing to enabling individuals with low self-esteem to access their internal resources more effectively. Cross-lagged study designs could also be employed to better examine the sustainability of the positive effects of self-affirmation. Jessop et al. (2023) also noted that it might be valuable for future studies to assess the short-term and long-term benefits of self-affirmation interventions. It might for instance be speculated that the focus on external resources is effective in the short-term (e.g., causes short-term increases in self-esteem) but that focusing on internal resources is more effective in the long-term (e.g., leads to long-term increases in self-esteem and well-being). Because most research has been conducted regarding beneficial long-term effects of self-affirmation with personal values in educational contexts (Easterbrook et al., 2021), this aspect remains to be further elucidated in future studies.

## 5 Conclusion

The first aim of the present study was to validate the Spontaneous Self-Affirmation Measure (SSAM). To achieve this, the original three-factor structure was independently validated on a relatively large sample with a wider age range. Second, we explored whether the SSAM can be integrated into a broader theoretical framework, that of resource activation. For this purpose, the existing factors of the SSAM were integrated into the taxonomy of resource activation. Specifically, the *Strengths* and *Values* factors were defined as an *Internal resource*s factor, and the *Social relations* factor as an *External resource*s factor. The results of confirmatory factor analyses showed that both the original 3-factor model and the complementary 2-factor model fit the data acceptably. All three first-order factors (*Strengths*, *Values* and *Social relations*, resp. *External resources* in the 2-factor model) and the higher-order factor *Internal resources* of the 2-factor model exhibited high reliability. Furthermore, it was demonstrated that the *Internal resources* factor is significantly more strongly correlated with self-esteem than the *External Resources* factor, which could have implications for theoretical considerations regarding the use of self-affirmation strategies, such as the Affirmational Resource View. We also found measurement invariance of the SSAM across across age, gender, and education which was tested with the 2-factor model. We propose that the results of the present study can also be extended to the clinical context. Resource activation is a common factor in psychotherapy and, as such, is an essential component in the treatment of various mental disorders (Grawe, 1999). The finding that individuals with high and low self-esteem appear to differ in their ability to spontaneously access their internal resources, while external resources seem to be relatively independent of self-esteem, may also have significant implications for the clinical context and resource activation in psychotherapy. In summary, the results of the present study underscore the validity of the SSAM and offer a complementary two-factorial structure of the SSAM that might help to link self-affirmation as a form of resource activation to more clinical contexts.

## Data availability statement

The datasets presented in this study can be found in online repositories. The names of the repository/repositories and accession number(s) can be found at: https://osf.io/62c3r/.

## Ethics statement

The studies involving humans were approved by Ethics committee of the University Hospital RWTH Aachen, Germany. The studies were conducted in accordance with the local legislation and institutional requirements. The participants provided their written informed consent to participate in this study.

## Author contributions

LR, BD, SF, VM, and SG have conceptualized the study. LR and LW conducted data analysis. LR wrote the first draft of the manuscript. BD, SF, LW, VM, and SG revised the manuscript. All authors contributed to the article and approved the submitted version.

## Footnotes

^1^In the sociodemographic questions, participants were asked to indicate their nationality. However, some people instead indicated their ethnicity.

^2^ https://qualtrics.com/

^3^Please note that the HIPT was not assessed because the differentiation between natural self-affirmation and regular positive thinking was already done by Harris et al. (2019) and fell out of scope of the current study.

^4^Please note that the grouping of the participants into ethnic groups was only possible because some participants identified their ethnic background as their nationality. Due to the small group size, the results should be interpreted with caution.

## References

[ref1] Adams G. Tormala T. T. O’Brien L. T. (2006). The effect of self-affirmation on perception of racism. J. Exp. Soc. Psychol. 42, 616–626. doi: 10.1016/j.jesp.2005.11.001

[ref2] Alicke M. D. Sedikides C. (2009). Self-enhancement and self-protection: what they are and what they do. Eur. Rev. Soc. Psychol. 20, 1–48. doi: 10.1080/10463280802613866

[ref3] Aronson J. Cohen G. Nail P. R. (2019). “Self-affirmation theory: an update and appraisal” in Cognitive dissonance: Reexamining a pivotal theory in psychology. ed. Harmon-Jones E. (Washington, DC: American Psychological Association), 159–174.

[ref6] Beyene K. Bekele S. A. (2016). Assessing univariate and multivariate homogeneity of variance: a guide for practitioners. Math. Theory Model. 6, 13–17.

[ref7] Bühner M. (2011). Einführung in die test-und fragebogenkonstruktion. München: Pearson Deutschland GmbH.

[ref9001] Burson A. Crocker J. Mischkowski D. (2012). Two types of value-affirmation: Implications for self-control following social exclusion. Soc. Psychol. Pers. 3, 510–516.

[ref8] Byrne B. M. (2010). Structural equation modeling with AMOS: Basic concepts, applications, and programming: Multivariate applications series. New York: Taylor and Francis Group.

[ref9] Cai H. Sedikides C. Jiang L. (2013). Familial self as a potent source of affirmation: Evidence from China. Soc. Psychol. Pers. Sci. 4, 529–537. doi: 10.1177/1948550612469039

[ref10] Cavanaugh J. E. Neath A. A. (2019). The Akaike information criterion: Background, derivation, properties, application, interpretation, and refinements. WIREs Comput Stat. 2019:e1460. doi: 10.1002/wics.1460

[ref11] Chen H. Li J. Zeng M. Yang J. (2021). Neural decoding of positive and negative self-knowledge. Motiv. Emot. 45, 585–598. doi: 10.1007/s11031-021-09900-7

[ref12] Cheung G. W. Rensvold R. B. (2002). Evaluating goodness-of-fit indexes for testing measurement invariance. Struct. Equ. Model. 9, 233–255. doi: 10.1207/S15328007SEM0902_5

[ref13] Cohen G. L. Garcia J. Apfel N. Master A. (2006). A self-affirmation intervention to reduce the racial achievement gap. science 313, 1307–1310. 16946074 10.1126/science.1128317

[ref14] Cohen G. L. Sherman D. K. (2014). The psychology of change: self-affirmation and social psychological intervention. Annu. Rev. Psychol. 65, 333–371. doi: 10.1146/annurev-psych-010213-115137, PMID: 24405362

[ref15] Creswell J. D. Dutcher J. M. Klein W. M. Harris P. R. Levine J. M. (2013). Self-affirmation improves problem-solving under stress. PLoS One 8:e62593. doi: 10.1371/journal.pone.0062593 23658751 PMC3641050

[ref16] Creswell J. D. Welch W. T. Taylor S. E. Sherman D. K. Gruenewald T. L. Mann T. (2005). Affirmation of personal values buffers neuroendocrine and psychological stress responses. Psychol. Sci. 16, 846–851. doi: 10.1080/15298860600805325 16262767

[ref17] Diedenhofen B. Musch J. (2015). Cocor: a comprehensive solution for the statistical comparison of correlations. PLoS One 10:e0121945. doi: 10.1371/journal.pone.0121945, PMID: 25835001 PMC4383486

[ref18] Düring C. Jessop D. C. (2015). The moderating impact of self-esteem on self-affirmation effects. Br. J. Health Psychol. 20, 274–289. doi: 10.1111/bjhp.12097, PMID: 24674228

[ref19] Easterbrook M. J. Harris P. R. Sherman D. K. (2021). Self-affirmation theory in educational contexts. J. Soc. Issues 77, 683–701. doi: 10.1111/josi.12459

[ref20] Emanuel A. S. Howell J. L. Taber J. M. Ferrer R. A. Klein W. M. Harris P. R. (2018). Spontaneous self-affirmation is associated with psychological well-being: evidence from a US national adult survey sample. J. Health Psychol. 23, 95–102. doi: 10.1177/1359105316643595, PMID: 27160152

[ref21] Ferrer R. A. Cohen G. L. (2019). Reconceptualizing self-affirmation with the trigger and channel framework: lessons from the health domain. Personal. Soc. Psychol. Rev. 23, 285–304. doi: 10.1177/1088868318797036, PMID: 30295141

[ref22] Flora D. B. (2020). Your coefficient alpha is probably wrong, but which coefficient omega is right? A tutorial on using r to obtain better reliability estimates. Adv. Methods Pract. Psychol. Sci. 3, 484–501. doi: 10.1177/2515245920951747

[ref23] Fox K. J. Harris P. R. Jessop D. C. (2017). Experimentally manipulated self-affirmation promotes reduced alcohol consumption in response to narrative information. Ann. Behav. Med. 51, 931–935. doi: 10.1007/s12160-017-9912-2 28484982

[ref24] Goldbach N. Reif A. Preuss H. Erhart M. Zabel K. Windmann S.. (2023). A randomized trial of group therapy for resource activation for patients with affective disorder. J. Psychother. Integr. 33:1:19. doi: 10.1037/int0000274

[ref25] Golino H. Shi D. Christensen A. P. Garrido L. E. Nieto M. D. Sadana R.. (2020). Investigating the performance of exploratory graph analysis and traditional techniques to identify the number of latent factors: a simulation and tutorial. Psychol. Methods 25, 292–320. doi: 10.1037/met0000255, PMID: 32191105 PMC7244378

[ref26] Graham J. M. (2006). Congeneric and (essentially) tau-equivalent estimates of score reliability: what they are and how to use them. Educ. Psychol. Meas. 66, 930–944. doi: 10.1177/0013164406288165

[ref27] Grawe K. (1997). Research-informed psychotherapy. Psychother. Res. 7, 1–19.

[ref28] Grawe K. (1999). Reasons and proposals for a general psychotherapy. Psychotherapeut 44, 350–359. doi: 10.1007/s002780050190

[ref29] Harris P. R. Griffin D. W. Napper L. E. Bond R. Schüz B. Stride C.. (2019). Individual differences in self-affirmation: distinguishing self-affirmation from positive self-regard. Self Identity 18, 589–630. doi: 10.1080/15298868.2018.1504819

[ref30] Harris P. R. Richards A. Bond R. (2023). Individual differences in spontaneous self-affirmation and mental health: relationships with self-esteem, dispositional optimism and coping. Self Identity 22, 351–378. doi: 10.1080/15298868.2022.2099455

[ref31] Hecke T. V. (2012). Power study of anova versus Kruskal-Wallis test. J. Stat. Manag. Syst. 15, 241–247. doi: 10.1080/09720510.2012.10701623

[ref32] Hepper E. G. Gramzow R. H. Sedikides C. (2010). Individual differences in self-enhancement and self-protection strategies: an integrative analysis. J. Pers. 78, 781–814. doi: 10.1111/j.1467-6494.2010.00633.x, PMID: 20433637

[ref33] Holmgreen L. Tirone V. Gerhart J. Hobfoll S. E. (2017). “Conservation of resources theory: Resource caravans and passageways in health contexts” in The handbook of stress and health: A guide to research and practice. eds. Cooper C. Quick J. C. (New York: John Wiley & Sons), 443–457.

[ref34] Hu L. Bentler P. M. (1999). Cutoff criteria for fit indexes in covariance structure analysis: conventional criteria versus new alternatives. Struct. Equ. Model. Multidiscip. J. 6, 1–55. doi: 10.1080/10705519909540118

[ref35] Jackson D. L. Gillaspy J. A. Jr. Purc-Stephenson R. (2009). Reporting practices in confirmatory factor analysis: an overview and some recommendations. Psychol. Methods 14, 6–23. doi: 10.1037/a0014694, PMID: 19271845

[ref36] Jessop D. C. Harris P. R. Gibbons T. (2023). Individual differences in spontaneous self-affirmation predict well-being. Self Identity 22, 247–275. doi: 10.1080/15298868.2022.2079711

[ref37] Kang H. (2021). Sample size determination and power analysis using the G* power software. J. Educ. Eval. Health Prof. 18:17. doi: 10.3352/jeehp.2021.18.17, PMID: 34325496 PMC8441096

[ref38] Kline R. B. (2011). Principles and practice of structural equation modeling. 3rd Edn. New York: Guilford Publications.

[ref39] Korkmaz S. Göksülük D. Zararsiz G. (2014). MVN: an r package for assessing multivariate normality. R J. 6:31. doi: 10.32614/RJ-2014-03126835159

[ref40] Łakuta P. (2023). Brief self-affirmation intervention for adults with psoriasis for reducing anxiety and depression and boosting well-being: Evidence from a randomized controlled trial. Psychol. Med. 53, 2574–2584. doi: 10.1017/S0033291721004499 34736546 PMC10123834

[ref41] Lannin D. G. Bible J. Harris P. R. Russell L. T. Arellanes J. A. Yazedjian A. (2021). How is spontaneous self-affirmation linked to self-esteem? A cross-lagged examination. J. Res. Pers. 92:104091. doi: 10.1016/j.jrp.2021.104091

[ref42] Leary M. R. (2007). Motivational and emotional aspects of the self. Annu. Rev. Psychol. 58, 317–344. doi: 10.1146/annurev.psych.58.110405.085658 16953794

[ref43] Martens A. Johns M. Greenberg J. Schimel J. (2006). Combating stereotype threat: The effect of self-affirmation on women’s intellectual performance. J. Exp. Soc. Psychol. 42, 236–243. doi: 10.1016/j.jesp.2005.04.010

[ref44] Martin P. (2002). Individual and social resources predicting well-being and functioning in the later years: Conceptual models, research and practice. Ageing Int. 27, 3–29.

[ref45] Maydeu-Olivares A. (2017). Maximum likelihood estimation of structural equation models for continuous data: standard errors and goodness of fit. Struct. Equ. Model. Multidiscip. J. 24, 383–394. doi: 10.1080/10705511.2016.1269606

[ref46] McQueen A. Klein W. M. (2006). Experimental manipulations of self-affirmation: A systematic review. Self Identity 5, 289–354.

[ref47] Nakao T. Miyatani M. Nakao M. Takezawa T. Maruishi M. Muranaka H.. (2009). Does medial prefrontal cortex activity during self-knowledge reference reflect the uniqueness of self-knowledge? 1. Jpn. Psychol. Res. 51, 69–84. doi: 10.1111/j.1468-5884.2009.00390.x

[ref48] National Research Council. (2003). Engaging schools: Fostering high school students’ motivation to learn. Washington, DC: National Academies Press.

[ref49] Pandey R. Tiwari G. K. Rai P. K. (2023). Understanding the efficacy of self-affirmation intervention for subclinical depression among young adults. Pract. Clin. Psychol. 11, 23–34. doi: 10.32598/jpcp.11.1.847.1

[ref50] Pietersma S. Dijkstra A. (2012). Cognitive self‐affirmation inclination: An individual difference in dealing with self‐threats. Br. J. Soc. Psychol. 51, 33–51. doi: 10.1348/014466610X533768 22435845

[ref51] Rashid T. (2015). Positive psychotherapy: A strength-based approach. J. Posit. Psychol. 10, 25–40. doi: 10.1080/17439760.2014.920411

[ref52] Rogers T. B. Kuiper N. A. Kirker W. S. (1977). Self-reference and the encoding of personal information. J. Pers. Soc. Psychol. 35, 677–688. doi: 10.1037/0022-3514.35.9.677, PMID: 909043

[ref54] Rosenberg M. (1965). *Rosenberg self-esteem scale (RSE)*. Acceptance and Commitment Therapy. Measures Package, No. 61. p. 18.

[ref55] Salgado S. González-Suhr C. Nazar G. Alcover C. M. Ramírez-Vielma R. Bustos C. (2022). Relationships between Individual and Social Resources, Anxiety and Depression in the Early Lockdown Stage by the COVID-19 in Chile. Behav. Sci. 12:357. doi: 10.3390/bs12100357 36285926 PMC9598173

[ref56] Savalei V. Reise S. P. Vazire S. Fried E. (2019). Don’t forget the model in your model-based reliability coefficients: a reply to McNeish. Collabra 5:36. doi: 10.1525/collabra.247

[ref57] Seaman E. L. Robinson C. D. Crane D. Taber J. M. Ferrer R. A. Harris P. R.. (2021). Association of Spontaneous and Induced Self-Affirmation with Smoking Cessation in users of a Mobile app: randomized controlled trial. J. Med. Internet Res. 23:e18433. doi: 10.2196/18433, PMID: 33666561 PMC7980123

[ref58] Sherman D. K. Cohen G. L. Nelson L. D. Nussbaum A. D. Bunyan D. P. Garcia J. (2009). Affirmed yet unaware: exploring the role of awareness in the process of self-affirmation. J. Pers. Soc. Psychol. 97:745. doi: 10.1037/a0015451 19856999

[ref59] Shi D. Maydeu-Olivares A. (2020). The effect of estimation methods on SEM fit indices. Educ. Psychol. Meas. 80, 421–445. doi: 10.1177/0013164419885164, PMID: 32425213 PMC7221491

[ref9002] Siegwart V. Schürch K. Benzing V. Roessler J. Everts R. (2022). Personal and social resources are linked to cognition and health-related quality of life in childhood cancer survivors. Children, 9:936. 35883920 10.3390/children9070936 PMC9322872

[ref60] Steele C. M. (1988). “The psychology of self-affirmation: sustaining the integrity of the self” in Advances in experimental social psychology. ed. Steele C. M., vol. 21 (Cambridge, MA: Academic Press), 261–302.

[ref61] Steele C. M. (1997). A threat in the air: how stereotypes shape intellectual identity and performance. Am. Psychol. 52, 613–629. doi: 10.1037/0003-066X.52.6.613, PMID: 9174398

[ref62] Steele C. M. Spencer S. J. Lynch M. (1993). Self-image resilience and dissonance: the role of affirmational resources. J. Pers. Soc. Psychol. 64, 885–896. doi: 10.1037/0022-3514.64.6.885, PMID: 8326471

[ref63] Symons C. S. Johnson B. T. (1997). The self-reference effect in memory: a meta-analysis. Psychol. Bull. 121:371. doi: 10.1037/0033-2909.121.3.371 9136641

[ref64] Tesser A. Crepaz N. Collins J. C. Cornell D. Beach S. R. (2000). Confluence of self-esteem regulation mechanisms: on integrating the self-zoo. Personal. Soc. Psychol. Bull. 26, 1476–1489. doi: 10.1177/01461672002612003

[ref65] Vandellen M. Knowles M. L. Krusemark E. Sabet R. F. Campbell W. K. McDowell J. E.. (2012). Trait Self–Esteem Moderates Decreases in Self–Control following Rejection: An Information–Processing Account. Eur. J. Pers. 26, 123–132. doi: 10.1002/per.1845 22611304 PMC3354773

[ref66] Voisin D. Brick C. Vallée B. Pascual A. (2019). When stereotype threat does not impair performance, self-affirmation can be harmful. Self Identity 18, 331–348. doi: 10.1080/15298868.2018.1454339

[ref67] Webb T. L. Benn Y. Chang B. P. Reynolds J. P. Sirois F. M. Assinnari A.. (2020). The relationship between individual differences in spontaneous self-affirmation and affect associated with self-weighing. J. Res. Pers. 89:104020. doi: 10.1016/j.jrp.2020.104020

[ref68] Zhou J. Li X. Tian L. Huebner E. S. (2020). Longitudinal association between low self‐esteem and depression in early adolescents: The role of rejection sensitivity and loneliness. Psychol. Psychother. 93, 54–71. doi: 10.1111/papt.12207 30488549

[ref69] Zhu X. Yzer M. (2021). Understanding self-affirmation effects: the moderating role of self-esteem. Br. J. Health Psychol. 26, 1016–1039. doi: 10.1111/bjhp.12517, PMID: 33641204

