# Supplementary material for: Internal and external self-affirmation resources: validation and assessment of psychometric properties of the spontaneous self-affirmation measure using structural equation modeling
Source: Front Psychol. 2024 Apr 4;15:1217416. doi: 10.3389/fpsyg.2024.1217416 (PMC11024277; doi:10.3389/fpsyg.2024.1217416)
Supplement: Supplementary file 1 [file Data_Sheet_1.docx]

**Internal and External Self-affirmation Resources: Validation and Assessment of Psychometric Properties of the Spontaneous Self-affirmation Measure using Structural Equation Modelling**

**Supplemental Material**

**Lena Rader^1*^ , Siegfried Gauggel^1^, Barbara Drueke^1^, Lorenz Weise^1^, Saskia Doreen Forster^1**^ & Verena Mainz^1**^**

^1^Institute of Medical Psychology and Medical Sociology, University Hospital RWTH Aachen, Pauwelsstraße 19, 52074 Aachen

*** Correspondence:**Corresponding Author
[lrader@ukaachen.de](mailto:lrader@ukaachen.de)

** these authors contributed equally

**Supplements index**

Supplements 1

Figure S1 - Factor model of the SSAM based on Harris et al. (2019) including a higher-order self-esteem factor

Supplements 2

Table S1 - Model fit of measurement models of classical test theory for the three first-order factors Strengths, Values and Social relations (resp. External resources) of the SSAM

Supplements 3

Table S2 – Results of the Levene’s test to test the assumption of homoscedasticity prior to conducting the analyses of variance

Table S3 – Parameters and results of the post-hoc power analyses of the ANOVAs for the Internal resources factor, External resources factor and the overall SSAM across age, gender, education and ethnicity

**Supplements 1**

**
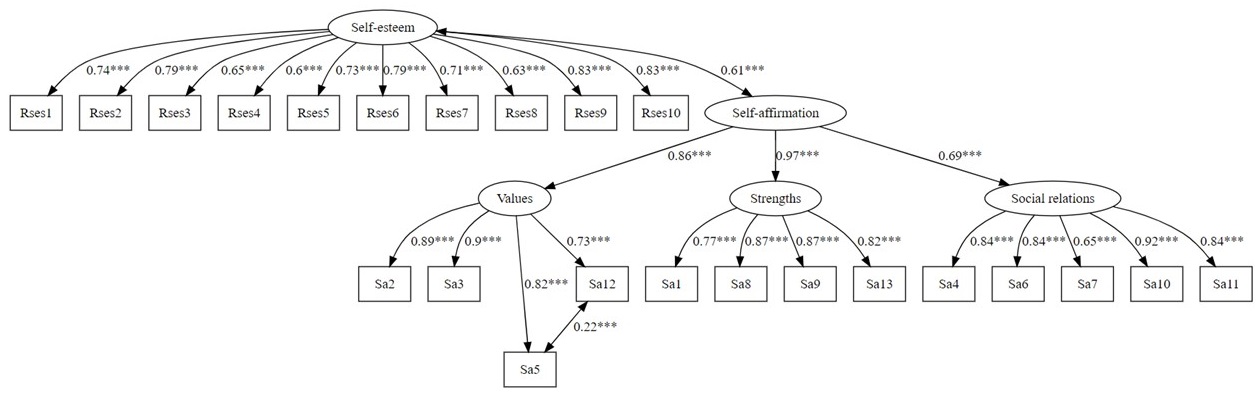
**

*Figure S1.* Factor model of the SSAM based on Harris et al. (2019) including a higher-order self-esteem factor

**Supplements 2**

Table S1

*Model fit of measurement models of classical test theory for the three first-order factors Strengths, Values and Social relations (resp. External resources) of the SSAM*

| *Factor* | *Measurement model* | $\chi^{2}$ | $df$ | $CFI_{s}$ | *RMSEA_s_* | *SRMR* | *AIC* |
| --- | --- | --- | --- | --- | --- | --- | --- |
| Strengths | Parallel | 268.33* | 11 | .772 | .146 | .087 | 14545.21 |
|  | Essentially parallel | 77.82* | 7 | .937 | .096 | .048 | 14312.68 |
|  | Tau-equivalent | 214.33* | 8 | .817 | .153 | .095 | 14418.29 |
|  | Essentially tau-equivalent | - | 4 | - | - | .054 | 14205.42 |
|  | Tau-congeneric | 5.30 | 2 | .997 | .039 | .008 | 14180.88 |
| Values | Parallel | 185.29* | 6 | .795 | .165 | .078 | 10381.58 |
|  | Essentially parallel | 51.35* | 3 | .945 | .121 | .046 | 10237.14 |
|  | Tau-equivalent | 168.16* | 4 | .812 | .193 | .099 | 10312.18 |
|  | Essentially tau-equivalent | - | 1 | - | - | .068 | 10190.22 |
|  | Tau-congeneric | 0.00 | 0 | 1.000 | .000 | .000 | 10148.57 |
| Social relations (resp. External resources) | Parallel | 863.51* | 17 | .064 | .213 | .129 | 17961.67 |
|  | Essentially parallel | 236.47* | 12 | .752 | .130 | .066 | 17253.13 |
|  | Tau-equivalent | 392.76* | 13 | .580 | .163 | .181 | 17385.03 |
|  | Essentially tau-equivalent | - | 8 | - | - | .093 | 16957.55 |
|  | Tau-congeneric | 32.16* | 5 | .970 | .070 | .018 | 16875.15 |

*Note*. Please note that the information matrix could not be inverted and robust test statistics could not be estimated for the essentially tau-equivalent models, CFI_s_ = scaled Comparative Fit Index, RMSEA_s_ = scaled Root Mean Square Error of Approximation, SRMR = Standardized Root Mean Square Residual, AIC = Akaike Information Criterion, **p*<.001

**Supplements 3**

Table S2

*Results of the Levene’s test to test the assumption of homoscedasticity prior to conducting the analyses of variance*

|  |  | Internal resources | | External resources | | Overall SSAM | |
| --- | --- | --- | --- | --- | --- | --- | --- |
|  | *df* | *F* | *p* | *F* | *p* | *F* | *p* |
| Age | 2, 1097 | 1.69 | .184 | 1.25 | .285 | 0.95 | .385 |
| Gender | 1, 1095 | 0.39 | .530 | 0.34 | .555 | 0.49 | .483 |
| Education | 1, 1004 | 6.02 | **.014** | 1.33 | .247 | 1.79 | .181 |
| Ethnicity | 2, 180 | 1.27 | .282 | 0.47 | .620 | 0.99 | .370 |

*Note*. Df = degrees of freedom; F = F-statistic of Levene’s test, p = p-value of Levene’s test; significant p-values are marked in bold.

Table S3

*Parameters and results of the post-hoc power analyses of the ANOVAs for the Internal resources factor, External resources factor and the overall SSAM across age, gender, education and ethnicity*

|  |  | Internal resources | External resources | Overall SSAM |
| --- | --- | --- | --- | --- |
| Age | α error probability | 0.05 | | |
|  | Total sample size | 1100 | | |
|  | Number of groups | 3 | | |
|  | Effect size | 0.03 | 0.02 | 0.01 |
|  | Power (1-β) | 0.13 | 0.08 | 0.05 |
| Gender | α error probability | 0.05 | | |
|  | Total sample size | 1097 | | |
|  | Number of groups | 2 | | |
|  | Effect size | 0.08 | 0.07 | 0.02 |
|  | Power (1-β) | 0.65 | 0.53 | 0.08 |
| Education | α error probability | 0.05 | | |
|  | Total sample size | 1006 | | |
|  | Number of groups | 2 | | |
|  | Effect size | 0.01 | 0.03 | 0.04 |
|  | Power (1-β) | 0.06 | 0.15 | 0.24 |
| Ethnicity | Error probability | 0.05 | | |
|  | Total sample size | 183 | | |
|  | Number of groups | 3 | | |
|  | Effect size | 0.33 | 0.11 | 0.26 |
|  | Power (1-β) | 0.98 | 0.24 | 0.88 |

*Note*. Age groups: 18-34 years (n = 202), 35-49 years (n = 366), 50-65 years (n = 532); Education groups: high school degree (n = 562), university degree (n = 447); Ethnicity groups: Black (n = 26), Asian (n = 14), White (n = 143); Cohen’s f was used to estimate the effect size.
